# Village health work in the primary healthcare of Zimbabwe: Health workers’ perspectives

**DOI:** 10.1371/journal.pone.0326956

**Published:** 2025-08-25

**Authors:** Ofhani Munyai, Azwinndini G. Mudau, Ntsieni S. Mashau

**Affiliations:** Department of Public Health, University of Venda, Thohoyandou, South Africa; University of Limpopo, SOUTH AFRICA

## Abstract

Migration and poor performance of healthcare workers in Zimbabwe hinder primary healthcare delivery. This has compelled the country to exploit the potential of village health workers in primary healthcare. The Village Health Worker Strengthening Plan was never operationalised owing to unclear roles and limited coordination. The study aimed to explore the roles, challenges, and strategies as informed by setting and context to improve the effectiveness and efficiency of Village Health Workers in service delivery. The study was conducted in the Beitbridge district, Matabeleland South Province, Zimbabwe. A qualitative exploratory survey with semi-structured interview guides was administered to 36 participants, comprising Village Health Workers, nurses, environmental health practitioners, and medical doctors. Three main themes were analysed, and 15 subthemes were found. The role of Village Health Workers was to promote health, prevent and control disease, diagnose and treat minor ailments, and conduct community-based disease surveillance and referrals for complicated cases to primary healthcare facilities. Challenges were inadequate logistical supplies, allowances, knowledge and skills, mobility, and personal protective equipment. Optimal stocking of the medical and equipment supplies, improving allowances, community health integration into the healthcare system, embracing mobile health technology, capacity building, and supportive supervision were sub-themes on suggested strategies to improve service delivery. The study helped clarify the roles and challenges and suggested strategies by health service providers, as informed by empirical findings, to improve the effectiveness and efficiency of Village Health Workers in service delivery. It is recommended that needs assessments be conducted on village health work to enhance their capacity and support.

## Introduction

Delivering essential health care services through primary health care (PHC) is vital towards realising universal health coverage (UHC). However, this is hindered by unsustainable shortages, imbalances, and poor performance of healthcare workers [1]. The World Health Organisation (WHO) reported that in 2013, African and Asian countries were the most affected, with a combined health worker shortage of 4.2 million [2]. In Zimbabwe, ‘*brain drain’*, HIV/ AIDS, and migration were often cited as the major contributory factors for healthcare worker shortages [3].

The Global Strategy on Human Resources for Health (2030) stated that the healthcare worker shortage compels low-income countries to harness the potential of Community Health Workers (CHW) in their PHC systems [4]. These have been defined as lay healthcare workers who are selected by their local communities, receive standardised training outside the formal nursing and medical curricula, and perform essential health services [5]. The term CHW has often been used as an umbrella term across different settings and program contexts to refer to health surveillance assistants (HAS) in Malawi, lady health workers (LHW) in Pakistan, rural health motivators (Swaziland), village health workers (VHWs) in Zimbabwe and Nigeria among other names in various countries [6,7] Click or tap here to enter text..

The conference of Alma Ata in 1978 provided a radical declaration for countries to achieve UHC through PHC services, which are community-driven and equitable [8]. It has been shown that VHWs can play vital roles in enabling health systems to achieve significant progress in improved effectiveness and efficiency of PHC services through increasing access to preventive and promotive services, early diagnosis and treatment of minor ailments in the communities through task-shifting [9].

The economic challenges in the early 2000s led to the near collapse of the health system in Zimbabwe as healthcare workers left the country for Western Europe in search of better employment conditions. Zimbabwe had eight healthcare workers per 10,000 people in 2015, which is against the WHO’s recommendation of 23 per 10,000 people [10]. Poor outcomes have characterised the healthcare system, with the infant mortality rate (IMR) having increased from 53 per 1000 in 1992–56 per 1000 live births in 2016; and the maternal mortality rate (MMR) still high at 443 per 100 000, with a way too high about the SDG target of 70 per 100 000 [11]. Matabeleland South Province in Zimbabwe has the highest HIV prevalence of 21% (against the overall 14% in the Country), of which Beitbridge district contributes 75% of this proportion [7].

Zimbabwe has been obliged to rely on the services of VHWs to bridge the gap in human resources for health. The VHW program effectively supported the rural health centres and clinics for promotive, preventive, curative and rehabilitative services at a household level under the context of PHC [7]. While the role of VHWs in low-income countries is essentially on communicable disease prevention and control, there is a gradual shift towards the management of lifestyle health conditions and noncommunicable diseases such as diabetes and hypertension in high-income countries [12]. The situation analysis of the community health system recommended the renaissance of the program to improve its effectiveness, efficiency, and sustainability in order for it to continue to play supportive roles of promotive, preventive, minor curative, and essential rehabilitative services equitably [7].

The Government of Zimbabwe has called for the revitalisation of the VHW program to bridge the gap between the formal health system and the communities to improve PHC delivery [7,10]. Whilst the VHW Strengthening Plan and the Community Systems Strengthening Framework for Health were developed, these were never operationalised as unclarified roles, limited coordination and duplication of community health services by the VHWs on specific population groups with the others remaining underserved [13].

Role clarity for VHWs in Zimbabwe can be crucial to delivering effective and efficient PHC services that address health inequities and improve health outcomes for the rural and underserved communities [7,10] Click or tap here to enter text.. There has been a dearth of studies in Zimbabwe that explored and clarified the roles of VHWs in developing and validating strategies to improve PHC service delivery. This study aimed to examine the role of VHWs in Beitbridge district, Zimbabwe, and identify their challenges in developing strategies that are informed by setting and context to improve their effectiveness and efficiency in delivering PHC services.

## Materials and methods

### Study design

A qualitative exploratory survey was used to explore and describe the role of VHWs in providing PHC services. This design enabled an in-depth and comprehensive understanding of the roles of VHWs by probing the perspectives of different health service providers working with these cadres and obtaining their lived experiences on the challenges and suggestions on how to overcome them. This would eventually enable their contribution to developing strategies to improve the effectiveness and efficiency of primary health care provision by the VHWs [14].

### Study setting

The study was undertaken in the Beitbridge district of Matabeleland South Province, Zimbabwe, which is a border town with the Republic of South Africa. This district was purposively selected as it was one of the most burdened in the country in terms of maternal, child, and reproductive health (MCRH) and communicable diseases such as TB and HIV, which rely on VHWs to provide support on PHC services in terms of community outreach. The district has 15 rural and 6 urban Wards, 20 public healthcare facilities comprising 18 rural healthcare facilities, 1 clinic, and a referral hospital. Most people in rural areas rely on VHWs to provide PHC services due to a shortage of professional healthcare and professional healthcare workers in the clinics. The communities speak Venda, Ndebele, Shona, and Sotho. The Housing and Population Census of 2022 found that the district had a population of 152,574, comprising 94000 rural and 58574 urban inhabitants [7]. The age distribution is a bit heavy, with over 60% being between 0 and 35 years, which places a high demand on the need for PHC services in terms of MCRH.

### Study population and sampling

The target population comprised 442 health service providers in the Beitbridge district who qualified in the inclusion criteria as they had a role to play in community health service delivery and in the activities of VHWs. Regarded as key informants and purposively selected were the District Medical Officer of Health (DMO), District Nursing Officer (DNO), 119 Registered General Nurses (RGNs), 8 Community Nursing Officers (CNOs), 27 Environmental Health Practitioners (EHPs), and 286 VHWs, who were conveniently selected from the PHC facilities.

The DMO is the chief executive officer, accountable for planning, organising, managing, coordinating, and implementing all health-related programs, including VHW activities in the district. The DNO is responsible for assessing district health needs, planning and organising the implementation of in-service training needs for nurses and VHWs, and monitoring and evaluating health programs. Community nurses work closely with facility-based nurses and health centre committees and are also involved in recruiting VHWs. RGNs and EHPs frequently supervise the VHWs.

Regarding health facilities, the district had 19 PHCs from three clusters comprising central (urban), east and west and one referral hospital. Purposively selected for the study were one urban clinic and the hospital, while 2 PHC facilities were randomly selected from each of the east and west clusters.

The study participants were recruited such that the district hospital would have the DMO, DNO, and six CNOs interviewed. Each of the five clinics would also have two RGNs, an EHP, and five VHWs participating in the study.

### Data collection

Semi-structured in-depth interview guides were used to explore and probe the participants on the VHW roles, challenges encountered, and the suggested strategies to improve their effectiveness and efficiency in delivering PHC services per the research objectives. The interviews were conducted in the languages of participants’ preference, English, Ndebele, Shona, Venda, and Sotho, which are spoken in the Beitbridge district. The interview guides were first pre-tested on nine participants (two nurses, two environmental health practitioners and 5 VHWs) from a non-participating PHC facility in Beitbridge district. The clarity of questions in the interview guides was improved before final data collection.

The interview sessions were then individually conducted with 21 key informants and 15 VHWs, ranging from 19 to 53 minutes. All 36 participants had given written consent to participate in the study. Throughout the interview sessions, the researcher directly interacted with the participants to elicit their feelings, perceptions, and viewpoints through probing, and this helped determine and shape meanings that could be influenced by context and setting, as explained by [15]. The interviews were recorded using a Sony tape recorder after obtaining prior permission from the study participants.

### Data analysis

Data collected was transcribed verbatim in vernacular languages and translated into English. The audio extracts were exported to MAXQDA Version 24 software, which aided the coding and thematic analysis. A six-step process according to Braun and Clarke (2012) involving familiarisation, coding, theme generation, reviewing themes, defining and naming the themes, and writing in terms of base themes and sub-themes was used [16]. These are presented in the results section.

### Ethical considerations

This study obtained ethical clearance from the University of Venda Research Ethics Committee (Registration FHS/23/PH/11/0709) and the Medical Research Council of Zimbabwe (MRCZ A/3175). Permission was also sought from the Ministry of Health and Child Care through the Permanent Secretary and the District Medical Officer. All the study participants provided written informed consent. On the informed consent process, a thorough verbal explanation of the research aims and what it entails was provided for participants’ comprehension before the interviews and an opportunity to ask questions and seek clarification. We also documented the informed consent process and participants’ understanding. We ensured comprehension of informed consent by assessing participants’ knowledge through discussions and encouraged feedback to identify areas of confusion. Finally, we sought re-consent if changes could occur during the interviews. Participants’ rights were respected through autonomy and decision-making capacity, and confidentiality was ensured throughout the research. The participants were advised that findings would be disseminated through publications and presentations at research symposia.

### Trustworthiness

Various measures were followed to ensure the trustworthiness of the research findings. For credibility, OM maintained prolonged engagements with the participants during interviews to build on their trust in the research process. Debriefing was also done on some selected participants. AGM and NSM verified the audio tape records to ensure data collection and analysis robustness. A logical, documented, and audit-traceable research process was followed to enable the dependability of the findings. OM also followed a high level of objectivity and conducted the research without any presuppositions to ensure the research process could be audited by an independent reviewer. The authors also ensured the transferability of the research findings by providing a robust contextual and a sufficiently thick description to compare phenomenological instances in different settings.

## Results

### Characteristics of the study participants

In-depth interviews were conducted with 36 participants. Females were the majority, with a total of 25 (69.4%) participants while the males were 11 (30.6%), as both key informants and VHWs were dominated by the former gender. The DMO (1), DNO (1), 6 CNOs, 9 RGNs, and 4 EHPswere reached and interviewed as key informants, and 15 VHWs also participated in the study. Two (2) participants from the key informants were not interviewed as they were away from their workstations during the interviews. Data saturation was achieved after 21 interviews when no new ideas were generated from the discussions (20); however, these continued until 36 participants were interviewed. **Table 1** shows the characteristics of the interviewed study participants.

**Table 1:** Characteristics of study participants.

Respondent type	Beitbridge Central/urban Male (Female)	Beitbridge West Male (Female)	Beitbridge East Male (Female)	Totals Male (Female)
DMO, DNO and CHNs	3(5)	0(0)	0(0)	3(5)
RGNs and EHPs	2(3)	2(2)	1(3)	5(8)
VHWs	0(5)	2(3)	1(4)	3(12)
**Total**	**5(13)**	**4(5)**	**2(7)**	**11(25)**

### Themes arising from data analysis

As provided in the background, three main themes emerged in line with the study’s objectives: the roles played by VHWs, the challenges faced by VHWs, and suggested strategies for improving service delivery by VHWs. The main themes were further synthesised, with 15 emerging sub-themes in **Table 2**.

**Table 2:** Themes emerging from the study.

Major theme	Major theme
Role of VHWs in the provision of primary healthcare service	1. Health promotions 2. Disease prevention and control 3. Diagnosis and treatment of minor ailments 4. Community-based disease surveillance 5. Referrals for complicated health cases to primary healthcare facilities
Challenges faced by VHWs in the provision of primary healthcare services	1. Inadequate and inconsistent medical and equipment supply 2. Transport challenges 3. Limited supply of personal protective clothing and equipment 4. Inadequate knowledge and skills 5. Inadequate and irregular incentives
Suggested solutions to overcome the challenges faced by VHWs in primary healthcare delivery	1. Optimal stocking of medical supplies, personal protective clothing, and equipment 2. Improved allowances 3. Community health integration into the mainstream healthcare system 4. Embrace information and communication technology 5. Capacity building and supportive supervision

### Role of VHWs in PHC

The role of VHWs emerged as the major theme for the study, and it had at least five sub-themes, which are overlapping and generally cross-cutting. These included health promotions, disease prevention and control, community-based disease surveillance, and referring communities to health facilities for specialist care, diagnosis, and treatment of minor ailments.

#### Health promotions.

It was mentioned that VHWs are primarily involved in the process of enabling communities and individuals to exercise control over their health and well-being. This role was reportedly anchored on health education, particularly with advocacy for behaviour change for communities and individuals to adopt healthy habits and lifestyles. Activities mentioned by the participants were centred around promoting safe sex, maternal, child, and reproductive health (MCRH), and good personal and household hygiene. VHWs mentioned that they distribute and encourage condom use in the communities as a means to prevent and control the spread of sexually transmitted infections (STIs), unwanted pregnancies, and conception. VHWs also cited that they advocate for individuals and partners for voluntary counselling and testing (VCT) to know their HIV status so that they can make informed decisions.

Whilst VHWs stated that they distribute birth control pills, they also encourage women with young children to visit health facilities to be administered long-term birth control options such as implants to prevent unwanted pregnancies. VHWs identify expectant women and encourage them to register pregnancies with the local health facilities within 14 weeks of gestation. To promote safe maternal health, VHWs would conduct home visits, particularly to the first gravidas, and encourage them to visit PHC facilities for a minimum of 8 antenatal care (ANC) check-ups.

Participants explained that VHWs play an essential role in child health, popularly known as ‘*baby-clinic’,* where they conduct monthly growth monitoring for the under-fives in their villages. Here, VHWs would weigh under-fives and plot their weights on the ‘baby-clinic’ health cards to monitor growth patterns against the expected child-growth curve. They also use mid-upper arm circumference (MUAC) measuring tapes to determine potential acute incidences of malnutrition in the children. VHWs clarified that they can identify possible ‘*danger-signs’* on the growth patterns of children and then refer such cases to the PHC facilities for further assessment. In the baby-clinic sessions, VHWs mentioned that they would routinely educate and encourage the nursing mothers and caregivers on the importance of exclusive breastfeeding for the first 6 months and on the recommended nutrition during childhood as they aim to prevent food deficiency syndromes from manifesting.

Participants also mentioned that VHWs are involved in preventing childhood illnesses by mobilising the communities to ensure the under-fives are immunised against child-killer diseases during the expanded program on immunisation (EPI) programs provided by the Ministry of Health from time to time. They keep registers for all the under-fives in their villages, monitor their vaccination statuses, and make follow-ups at their homes. Health service providers also mentioned that VHWs were trained and participated in the administration of administering vitamin A supplementation and oral polio vaccines (OPVs).

Participants said:

*“We encourage exclusive breastfeeding for the first 6 months and to continue until the child reaches 18 months. We also educate mothers and caregivers on the recommended supplementary feeding”. (VHW, participant number 9)*

*“We always advocate for a 4-start infant diet, which is considered balanced. Such a diet should include carbohydrates, proteins, vitamins, and mineral salts. The most important component of this diet are proteins, particularly beans, as this helps prevent kwashiorkor, which is quite common in some poor communities”. (VHW, participant number 13)*

VHWs advocate for safe water, sanitation and hygiene practices in the villages, encouraging communities to construct *Blair ventilated improved pit* latrines (BVIP) with hand washing facilities for safe disposal of faecal matter and hand hygiene (respectively). For household and personal hygiene, the communities are also encouraged to use chlorine detergents to clean the toilets and have *veronica buckets* (a bucket with soapy water fixed with tape at the bottom) mounted at hand height and a container to collect wastewater. Participants mentioned that in the absence of the now commonly used Veronica buckets, VHWs advocate for the communities to install tippy tapes, which similarly serve the same function as the former. Through the efforts of VHWs, rural communities are also obligated to dig waste disposal pits where these would be covered daily with ash to control the breeding of houseflies. VHWs also mentioned inspecting the kitchens to check for cleanliness, safe water, and cooking utensils storage conditions.

One of the participants mentioned that:

*“During Wednesdays, our communities know they will likely be visited by a VHW. We will be advocating for a complete 5-point plan for household hygiene. A homestead should have a toilet with a hand washing facility, a waste disposal pit, a pot rack, and a clean kitchen. Where there is no toilet, we leave that homestead having pegged it, and where there is no hand wash tap, we ensure that a veronica bucket or a tippy tap is installed…We would then make ad-hoc follow-ups to check on the household hygienic practices.” (VHW participant, number 19).*

*“I always maintain a duty roaster for cleaning water points by the residents on a weekly basis. Moreso, practices such as washing clothes within 30m of the boreholes are strictly prohibited”. (VHW, Participant number 12).*

#### Diagnostic and curative services for minor ailments.

It was mentioned that VHWs complement the nurses through task shifting. It was stated that VHWs use rapid diagnostic test (RDT) kits in their local communities to diagnose malaria and are authorised to administer court drugs to manage it. Health service providers also acknowledged a high burden of diarrhea in the communities of Beitbridge and described it as a major cause of death among the under-fives. Participants mentioned that VHWs educate the communities in preparing sugar and salt solution (SSS) which they use to treat dehydration. Other minor ailments, such as wound dressing, which require first aid interventions, are managed at the community level by the VHWs, with the application of betadine being cited to prevent wound infection. Health service providers emphasised that VHWs should always have adequate stocks of pain relievers, rehydration salts, and anti-malaria drugs, which are essential as first-line medicines.

Participant said:

*“We educate our communities on how to prepare sugar and salt solutions to treat dehydration from diarrhoea. Its preparation would involve boiling clean water, letting it cool until it gets warm, and administering 6-level teaspoons of sugar and a half-level teaspoon of salt on a litre of warm water. The caregivers are encouraged to ensure the one suffering from diarrhea drinks the solution to avoid circulatory collapse…. This solution can be the difference between life and death…” (VHW, participant number 10)*

#### Referral services.

Most of the participants mentioned that VHWs link the local communities to the PHC facilities, thus providing a referral pathway. VHWs revealed that their communities consult them whenever there is an infirmity to assess the condition(s) and determine the need for referral to the local clinic. One community nurse stated they used to provide VHWs with patient referral slips, which the latter would endorse to indicate the intervention they would have administered (if any) and other information that might be of medical relevance.

Participant mentioned:

*“As VHWs, we assess for the signs and symptoms of health conditions and try to diagnose and treat if the condition seems minor. However, if we presume TB, we refer to the clinics. Similarly complicated cases of malaria are managed at the health facilities.” (VHW, participant number 13)*

#### Disease prevention and control.

VHWs are involved in preventing and controlling prevalent diseases in their communities. Malaria prevention and control was frequently mentioned because Beitbridge district was highly burdened. VHWs are involved in distributing insecticide-treated bed nets, and they routinely monitor their usage during their door-step health promotional activities. They also interact with their communities to identify and monitor mosquito breeding sites and report to the health facilities for control by the EHPs. VHWs encourage safe water, sanitation, and good hygiene practices by assisting the communities on proper siting of Blair toilets to prevent contamination of water sources with human excreta. During disease outbreaks and other public health emergencies, VHWs issue water treatment chemicals and educate the local communities on sustainable and economical household water treatment methods. VHWs help to control the spread of tuberculosis (TB) in their local communities by monitoring case adherence to directly observed therapy (DOT), tracing their close contacts, screening them (or referral to the clinic), and making constant follow-ups for possible manifestations of signs and symptoms of the disease. Fundamental to this role is health education on the signs and symptoms, as well as prevention and control of the spread of infection.

Participant said

*“When I get information about any member of my community having persistent cough with/without blood for more than a week, I visit their home, assess the signs and symptoms and refer them to the health facility for further assessment. I would also be eager to know the clinic’s outcome of such an assessment. They nicknamed me ‘class monitor’ in my village as I would regularly conduct home visits to check on the health status of the communities”. (VHW, participant number 22).*

#### Public health surveillance.

VHWs conduct community-based disease surveillance (CBS). The EHPs particularly mainly explained that VHWs are the initiators of a series of events leading to a coordinated response for any disease or unusual event of public health importance in the communities. VHWs stated that they first visit the homestead where there is a suspected case to confirm the rumour and deliver health education to prevent the possibility of further transmission of infection before reporting to the EHPs for verification. Health service providers mentioned that VHWs are the conduits of transmitting unusual and/or unstructured health-related events/diseases to inform the health facilities through a system they call ‘*signal*’ reporting, and this forms the basis for triggering systematic investigation for possible disease outbreaks. The VHWs also specified that they use ‘*Signal Reporting Forms’* to report events of importance to public health. In most instances, they travel to the health facility to report such cases, while in exceptional circumstances, they phone the nurse-in-charge of their clinic if they have airtime. VHWs appreciate that their local community entrusts them with responding quickly to the ‘signals to respond to ‘signals and cascades them to the formal health system.

Participants said:

*“…communities have always entrusted us to report unusual disease occurrences such as suspected COVID-19, malaria, or cholera cases in their localities. The communities know that we are always available for any health-related matter in the villages as we are the extension of the health system, and we regard this as a calling”. (VHW, participant number 11)*

*“We report unusual events/diseases in the form of signals to the nurse-in-charge of our local clinics. In most cases, we verify the incidences to prevent false alarms, we verify the incidences to prevent false alarms before cascading such reports to the nurse in charge…”. (VHW, participant number 26)*

*“VHWs contribute immensely to disease surveillance as they can detect outbreaks early to facilitate targeted interventions. We have managed to control COVID-19 successfully and, recently, cholera outbreaks with the help of VHWs. We regard them as valuable assets for the health system in the local communities in terms of public health surveillance…”. (Key informant, participant number 33)*

### Challenges faced by VHWs

Participants indicated that VHWs face numerous challenges when executing PHC services. These were synthesised along the following themes: inadequate and inconsistent medical and equipment supplies; limited personal protective clothing (including uniforms) and equipment; inadequate and inconsistent incentives and transport to reach far away and hard-to-reach areas; and inadequate knowledge and skills. These challenges were cited as a barrier to the delivery of effective and efficient community health programs in the district.

#### Inadequate and inconsistent medical and equipment supplies.

It was mentioned that VHWs should be supplied with well-stocked medical kits with essential medicines and equipment to enable them to provide basic healthcare services in remote settings. Key informants mentioned that they expect VHWs to be supplied with the following accessories: disposable latex gloves and masks, eye protection and gowns; essential medicines such as antimalaria antibiotics, pain relievers, vitamin A supplements, oral rehydration salts (ORS); rapid diagnostic kits for malaria, HIV and pregnancy; thermometers; first aid accessories such as bandages, wound dressings and antiseptic solutions; baby growth monitoring equipment such as baby weighing scales and MUAC-measuring tapes. Other participants also mentioned water purification tablets, condoms, family planning pills, information education and communication (IEC) materials, and stationery such as recording and reporting tools such as Registers, Forms, and pens. However, it was revealed that VHW’s medical kits were less than 20% in terms of stocking levels as the system for replenishment is no longer followed. The district hospital has persistently understocked with essential medicines since the early 2000s.

Participants said:

*“It hurts me having to see fellow community members in pain and having to travel long distances of over 15km to the health facility without having administered to them medication to manage pain”. (VHW, participant number 6).*

*“The communities sometimes lose confidence in our relevance as we fail to provide curative services for minor ailments and us having to refer them to the clinics… Our communities are now regarding us as thieves who steal medicines as they could not believe that we do not have pain-relievers in our stocks”. (VHW, participant 22)*

*“Three VHWs are made to share a single scale and a MUAC-tape for use in our respective villages. We have operational challenges as we live far away from each other…”. (VHW, participant 11)*

*“I am expected to submit 7 Registers every month at my clinic, and it has been a long time since we receive such stationery. We use our financial resources to buy exercise books, which we then improvise to make registers. This is taking a strain on our livelihoods”. (VHW, Participant 8)*

*“… as you are aware our healthcare system has nearly collapsed since the early 2000 and has not fully improved; we cannot expect the VHWs to be supplied with essential medicines when the referral hospital does not even have paracetamol! Our health system financing is mainly out of pocket expenditure…”. (Key Informant, participant number 3)*

#### Transport challenges.

Participants mentioned that bicycles are not consistently supplied to the VHWs to conveniently travel in the communities when conducting health-related duties. Some VHWs cited that they were last issued with such equipment over 5 years ago, and these are now obsolete since there is no provision for their servicing and/or replacement of worn-out parts. Other participants exposed that they have never been supplied with bicycles since they started practicing as VHWs over 5 years ago. They walk long distances and sometimes rugged terrains when executing their duties. It was revealed that the Ministry of Health relies on donors to supply bicycles to the VHWs.

Participants said:

*“I often receive calls from far away from my homestead, and without a bicycle, I go there on foot to assess the situation…. Sometimes at night, we respond to such ‘signals’ without any portable lighting, and we fear getting attacked or even killed by dangerous wild animals”. (VHW, participant number 10)*

*“I walk close to 12 km to the clinic to present my monthly report, which has strained my body, especially considering my old age. My bicycle is no longer in good condition as I cannot afford to service it or replace worn-out parts routinely. My legs can no longer carry me that far, so I sometimes fail to report to the health facility every month as expected”. (VHW, participant number 9)*

#### Limited supply of personal protective clothing/equipment.

Latex gloves and face masks were reportedly in short supply for the VHWs with fears of getting exposed to infectious disease agents. From the interviews, it seems there is no system to resupply uniforms/ protective gowns and other personal protective equipment. Most of the VHWs complained that their uniforms were worn out. Some cadres stated that the uniforms were last supplied over 4 years ago and were issued without considering the respective VHWs’ anthropometry measurements. Others bemoaned that they felt stigmatised by wearing uniforms that could not fit their body sizes properly. VHWs appreciated that while they were supplied with sunhats to protect them against the sun’s rays during hot days, there are no rain courts to shield their bodies in rainy weather conditions.

Participants said:

*“We sometimes must endure harsh rainy weather conditions without raincoats. The ministry should supply us with such clothing as we are an extension of the health system”. (VHW, participant 9)*

*“Sometimes we have to handle patients without gloves and face masks! We are always exposed to infectious disease agents. We are surviving on God’s mercy”. (VHW, participant 13)*

#### Inadequate and irregular incentives.

Participants mentioned that the healthcare system in Zimbabwe recognises VHWs as volunteers, and these are given a quarterly allowance of US$42. Participants indicated that they do not receive their allowances consistently, contributing to most VHWs losing morale to diligently execute their duties. Considering the demand of work that VHWs go through in their villages, key informants and VHWs unanimously concurred with each other that the allowance currently paid to the latter is no longer serving many purposes, considering the evolution of the role of VHWs in PHC, which has incorporated task shifting.

Participants said:

*“It can take up to 6 months to get the US$42 allowance, which we were advised that we shall receive by the end of 3 months. We appreciate that we are volunteers, but they should give us our allowance well on time so that we can buy soap to wash our single uniforms”. (VHW, participant number 14)*

*“Today, we talk of task shifting, meaning that VHWs are more involved in the diagnostic and curative services to complement the registered general nurses. The quarterly allowance of US$42 is not enough for these cadres. The Ministry of Health should consider increasing this amount to motivate VHWs”. (Key Informant, participant number 1)*

#### Inadequate knowledge and skills.

The health service providers indicated that there seems to be inadequate knowledge and skills for the majority of VHWs in the district on new therapeutic developments in the medical field, which some attribute to inadequate refresher training. It was mentioned that when training programs exist, only a few VHWs are invited to such programs. The ones not taking part might not improve their knowledge and skills in managing certain health conditions. Some of the respondents indicated that most of the training programs have allowances. Those not in attendance lose an opportunity to realise some monetary incentives, which contributes close a chance to realize some monetary incentives, contributing to the loss of morale for the job.

### Suggested strategies to improve service delivery by VHWs

Most of the suggested strategies to improve the effectiveness and efficiency of VHWs in service delivery were proffered in line with resolving the challenges faced by these cadres. The subthemes included the following: putting a system to ensure optimal stocking and monitoring of the medical and PPE supplies for VHWs; mobility improvements; incorporating mobile health technology in VHWs programs; integration of VHWs into the mainstream health system; and conducting regular refresher training.

#### Optimal stocking of the medical supplies and PPE for VHWs.

It was mentioned that the Ministry of Health’s community health guidelines provided a checklist for the required stocks of medical supplies. In line with task shifting, key informants have indicated that there should be improved healthcare funding for essential medical supplies to ensure improved accessibility to the local communities through the VHWs. Some key informants also pointed out that adequate stocks for VHWs’ medical supplies would help decongest the local health facilities since minor ailments would be managed at the community level. It was also suggested that there should be a system that tracks the medicine usage by the VHWs to promote accountability. To protect VHWs from infectious disease agents, adequate PPE should be supplied, such as gloves, gowns, face masks, and uniforms. Some participants suggested that VHWs should be supplied with uniforms at least annually. At the same time, other respondents were of the view that they should be given uniform allowances to purchase for themselves.

#### Improve on mobility.

Regarding mobility for the VHWs to efficiently reach far away areas from their homesteads to deliver PHC services, it was suggested that bicycles should be supplied at least once in two years. Some participants also recommended that the Ministry of Health put a system for quarterly maintenance and repair of such pieces of equipment. One key informant even suggested that motorbikes be supplied to the best-performing VHW cadres to incentivise good service delivery performance.

#### Community health integration into the mainstream health system.

Most of the key informants appreciated the evolution of the role played by VHWs in task shifting from primarily health education to diagnostic and curative services. In line with this, there were suggestions that VHWs be incorporated into the mainstream healthcare system rather than regarded as volunteers. This was qualified to boost their morale in PHC service delivery and provide them with opportunities for career advancement in the health sector. It was also imagined that VHWs’ integration into the healthcare system could improve their remuneration. Participants emphasised that the healthcare system would not function appropriately without the involvement of the VHWs as these are a link with the communities and are vital in terms of surveillance of diseases and other public health events.

#### Use of mobile health technologies.

Some key informants have suggested using mobile health (mHealth) technologies, including smartphones and tablets, to improve VHW service delivery. Participants clarified that in addition to using paperwork, mHealth would enable remote data collection using mobile devices. Key informants explained that mobile devices could improve the effectiveness and efficiency of VHWs in service delivery through having easy access to electronic health information, guidelines and reference materials, thus enabling them to make informed decisions. It was further elaborated that mHealth could improve monitoring and real-time reporting of health-related data through facilitating, coordinating, and collaborating with other VHWs, healthcare workers, and stakeholders.

#### Capacity building and supportive supervision.

Health service providers have raised the concern that some VHWs lack adequate skills to diagnose and manage some of the ailments they are expected to diagnose and/or operate at the community level. Participants suggested that the Ministry of Health should provide a program for refresher training and supportive supervision for VHWs, particularly to equip them with knowledge and skills on new advancements in diagnostic and treatment procedures for certain emerging/re-emerging infirmities. Here, there was a divergence of opinions among the health service providers, with some suggesting that such refresher training programs be made at the local clinic level, while others opted for district-level planning. Supportive supervision for these cadres was also recommended to provide mentorship for the VHWs.

## Discussions

This study validated the universal roles of VHWs, which include health promotion, disease prevention and control, diagnostic and treatment of minor ailments, health education and advocacy, referrals, and community mobilisation on health-related issues and interventions [17]. VHWs play a vital role in both developed and developing countries, but their focus differs due to varying healthcare systems, disease burden, resource availability, and health needs [18] The functions of CHWs can be custom-made to address specific local health priorities in various settings [19]. Beitbridge reportedly focuses on delivering essential healthcare services, emphasising infectious disease prevention and control and MCRH, which is consistent with the findings in the Mudzi and Mutoko districts of Zimbabwe [10].

For the health promotion role, VHWs in Beitbridge put much prominence on educating the communities on MCRH with advocacy for early pregnancy registration for ANC check-ups and exclusive breastfeeding for the first 6 months; They continued until 18–24 months and a balanced diet for the infants. Like in many developing countries, Zimbabwe is highly burdened with an infant mortality rate of 56 per 1000 live births against an SDG target of 70 per 100000 [11]. This was also consistent with studies in Mali and Malawi, where under-five mortality is a critical challenge for the healthcare systems due to a shortage of healthcare professionals compelling CHWs enrolled to assist in rural settings for MCRH promotion [18]. A study in rural South Africa by Le Roux has shown improved MCRH outcomes, such as more ANC visits and better baby feeding practices, attributable to CHWs conducting home visits [20].

This study found that VHWs in Beitbridge were mostly concerned with the prevention and control of infectious diseases such as malaria, HIV, and TB, as might be compelled by a high burden in that district. In 2015, an HIV prevalence of 21%(7) a TB incidence rate of 210 per 100,000 population in 2019 [21] and 79% of its population at risk of contracting malaria in 2017 [22]. This probably justifies so many prevention and control efforts on these infectious diseases. There was not much mention of the management of non-communicable diseases by health service providers or the VHWs despite the double burden of communicable and non-communicable diseases faced by the country and other developing countries [21]. (12)It was acknowledged that while the CHW role is widely understood in MCH and infectious disease prevention and control, new roles include diabetes and other lifestyle diseases [12]. Like in high-income countries, the revitalisation of the PHC program in South Africa has started to utilise CHWs in non-communicable disease control programs. CHWs monitor blood glucose levels, blood pressure, and potential complications, including social support for the affected families [12].

The study has shown that VHWs are involved in the distribution of condoms and only refer women of reproductive age to health facilities for other options for family planning, such as birth control pills and injectables. Over 60% of the women in sub-Saharan Africa who need modern contraceptives are disproportionately affected by having to find trained physicians to operate them in health facilities [23]. Modern contraception, such as injectables and pills, was found to be more effective in enabling child spacing. Improving access to such family planning methods could be essential in safeguarding the well-being of women and children. The health systems of Nigeria, Madagascar, Rwanda, and Uganda, among others, have, through task-shifting, trained CHWs to administer injectable contraceptives, albeit under consistent supervision with monitoring and evaluation of such programs [23]. In these countries, studies have demonstrated a higher prevalence of injectable contraceptive uptake and comparable quality outcomes among the women exposed to CHWs as compared to those in facility-based settings [23]. In Rwanda, injectable family planning uptake has increased from less than 20% to over 50% between 2005 and 2015 through the integration and capacitation of CHWs [24].

This study revealed that VHWs play an essential role in public health surveillance where unusual events referred to as ‘*signals’* are followed up and verified in collaboration with local community involvement. As was mentioned in the study, the district continues to be affected by intermittent disease outbreaks such as cholera, and VHWs assist in identifying and containing outbreaks before spreading to the larger population. This finding is consistent with a scoping review of 25 low and middle-income countries by Alhassan and Wills [25]. who reported that CHWs contribute to public health surveillance through patient follow-ups and contact tracing, where community engagement, data gathering, screening, testing, and health education are integral to the program [25].

The study findings revealed little or no mention of VHWs’ use of ICT in their routine practice and/or reporting public health-related events. This could lead to delays in the real-time communication of health information to the facilities. A study by [26] Tanzania also revealed limited use of mobile health technology during the capturing and transmitting public health surveillance data and reports to health facilities. This has contributed to delays of nearly a week in relaying health-related information to the facilities [26] Public health surveillance entails early warning systems in terms of detection, reporting, and coordinated response, which could be improved by the use of ICT. Real-time recording and transmission of health-related data could enable the timely detection of potential disease outbreaks and inform response planning.

VHWs in Beitbridge mentioned challenges in terms of stocking essential medicines for the treatment of minor ailments in the communities, and this was consistent with findings by [10,27] where malaria drugs were reported to be consistently undersupplied, thus hampering case management at the community level, studies have shown that VHW services could be improved by using ICT to monitor and manage medicine stock levels. In Malawi, an ICT program known as *cStock* enables the Health Surveillance Assistants (HSAs) to send short-message-service (SMS) on medicines stocking levels where they interact with a dashboard operated at the district level, which enables them to get updates as to when they can replenish their medical stocks [26].

The study has shown the traditional use of measuring aids like MUAC tapes by VHWs for growth monitoring for under-five children. Mobile health (mHealth) technology can also be incorporated to complement and improve the monitoring of children’s possible danger signs. In Malawi, mHealth was integrated into community case management (iCCM) applications in mobile phones (tablets), where interactive checklists enabled HSAs to assess and manage children with symptoms of diarrhoea, cough, rapid breathing, and fever, among other danger signs. These applications have health guides, which enable the HAS to interact and quiz the mHealth application system on a series of health-related questions to diagnose and treat certain infirmities [28].

VHWs were reported to be playing an essential role in anchoring the healthcare system in Beitbridge. However, these cadres are regarded as volunteers with reports of waning motivation and increasing attrition. There were suggestions from key informants for integrating VHWs into the mainstream health system as this could lead to their increased motivation through recognition by communities, support and supervision from healthcare professionals, improved and consistent provision of incentives [29]. These submissions are consistent with the Family Health System of Brazil [30] CHWs’ integration into the Unified healthcare system improved PHC outcomes. A systematic review by Tshering et al. [31] revealed that individual, family, social, and organisational factors influenced VHWs’ motivation and retention. The review qualified that VHWs value healthcare system recognition in terms of improved financial and nonfinancial incentives, an enabling work environment, supportive supervision and mentorship [31,32] Click or tap here to enter text.. Integrating VHWs into the healthcare system was also reportedly effective in Nigeria. These cadres contributed to increased demand and utilisation of MCRH services through the Subsidy Reinvestment Program (SURE-P) [33]. It was also found that the critical success factors for the VHWs were linked to their motivation: a sense of identity, a feeling of confidence and being recognised by the healthcare system, and the expectation of valued outcomes [33].

### Limitations of the study

The study was conducted in one district of Zimbabwe, and its qualitative nature limits its ability to generalise the findings to the broader population. However, the themes generated present an opportunity for validation by a quantitative study.

## Conclusions

Village health workers (VHWs) in Beitbridge district play vital roles in PHC in terms of health advocacy in their local communities by promoting the consumption of safe water, good sanitation and hygiene practices, and safeguarding maternal, child, and reproductive health. They conduct health education to prevent and control infectious diseases like malaria, TB, and diarrhea. Village Health Workers use RDTs to diagnose and treat uncomplicated malaria cases and manage minor ailments such as diarrhea, wounds, and headaches. They help detect potential disease outbreaks through community-based surveillance activities. VHWs face numerous challenges in executing their tasks, such as inadequate and inconsistent logistical and equipment supplies and presumed inadequate knowledge and skills. Strategies suggested to improve the effectiveness and efficiency of VHWs in PHC delivery include optimal stocking of the medical supplies and PPE for VHWs, embracing the use of mobile health technologies, and integrating community health services into the mainstream healthcare system. It is recommended that the Ministry of Health and Child Care in Zimbabwe carry out a comprehensive and consultative needs assessment to identify gaps for VHW capacity building and program support.

## Supporting information

S1 File Inclusivity in global research questionnaire. (DOCX)
